# The Updated Genome of the Burying Beetle *Nicrophorus vespilloides*, a Model Species for Evolutionary and Genetic Studies of Parental Care

**DOI:** 10.1002/ece3.70601

**Published:** 2024-12-09

**Authors:** Christopher B. Cunningham, Kyle M. Benowitz, Allen J. Moore

**Affiliations:** ^1^ Department of Entomology University of Georgia Athens Georgia USA; ^2^ College of Integrative Sciences and Arts, Arizona State University Mesa Arizona USA

**Keywords:** coleoptera, Silphinae, social behavior, Staphylinidae

## Abstract

Understanding the evolution of social behavior requires establishing links between genomes and social phenotypes. High quality genomic resources from a diverse set of social species are required for both broad scale comparative genomic analyses and targeted functional genomic experiments and are therefore crucial for this goal. Here, we report on an updated genome for the burying beetle *Nicrophorus vespilloides* , an evolutionary and genomic model species for social behavior and parental care. The new assembly used PacBio sequencing reads and long read assemblers. This version of the genome greatly improves the continuity of the assembly and added new annotations, particularly lncRNA's. These updates will allow this resource to continue to be useful for newer functional genomic techniques. This improved assembly will also keep *N. vespilloides* a valuable comparative genomic resource. Updating genomic resources will continue to allow the field to make discoveries about the evolution of complex phenotypes, such as parental care.

## Graphical abstract

Social behavior is a seminal evolutionary innovation. To fully understand its evolution, its genomic underpinnings must be studied in a variety of organisms. We substantially updated the genome assembly and annotation of *Nicrophorus vespilloides* , a beetle that has elaborate parental care and has served as a model to understand the genetics of parental care. This updated resource will allow this species to continue to be used with increasing sophisticated functional genomic techniques and continue to be a resource for genomic studies of insects.

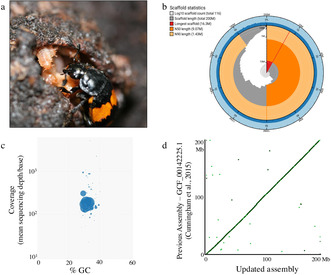

## 1 Introduction

A complete understanding of phenotypes can only be achieved by investigating broad scale evolutionary forces and the genomic substrate upon which those forces act (Hofmann et al. 2014). Social behavior presents a particular challenge because of its complexity and flexibility, and how multiple levels of biology are integrated to produce social traits (Boake et al. 2002; O'Connell and Hofmann 2011). Tackling this complexity requires behavioral models be chosen broadly, developed with high quality genomic resources, and studies to find core mechanisms controlling social behavior (Phelps et al. 2010; Hofmann et al. 2014). To that end, we developed genomic resources for the burying beetle *Nicrophorus vespilloides* , a beetle with elaborate parental care (Cunningham et al. 2015). Here, we report a significantly improved and updated *N. vespilloides* genome produced using PacBio data and long read assemblers.

**FIGURE 1:**
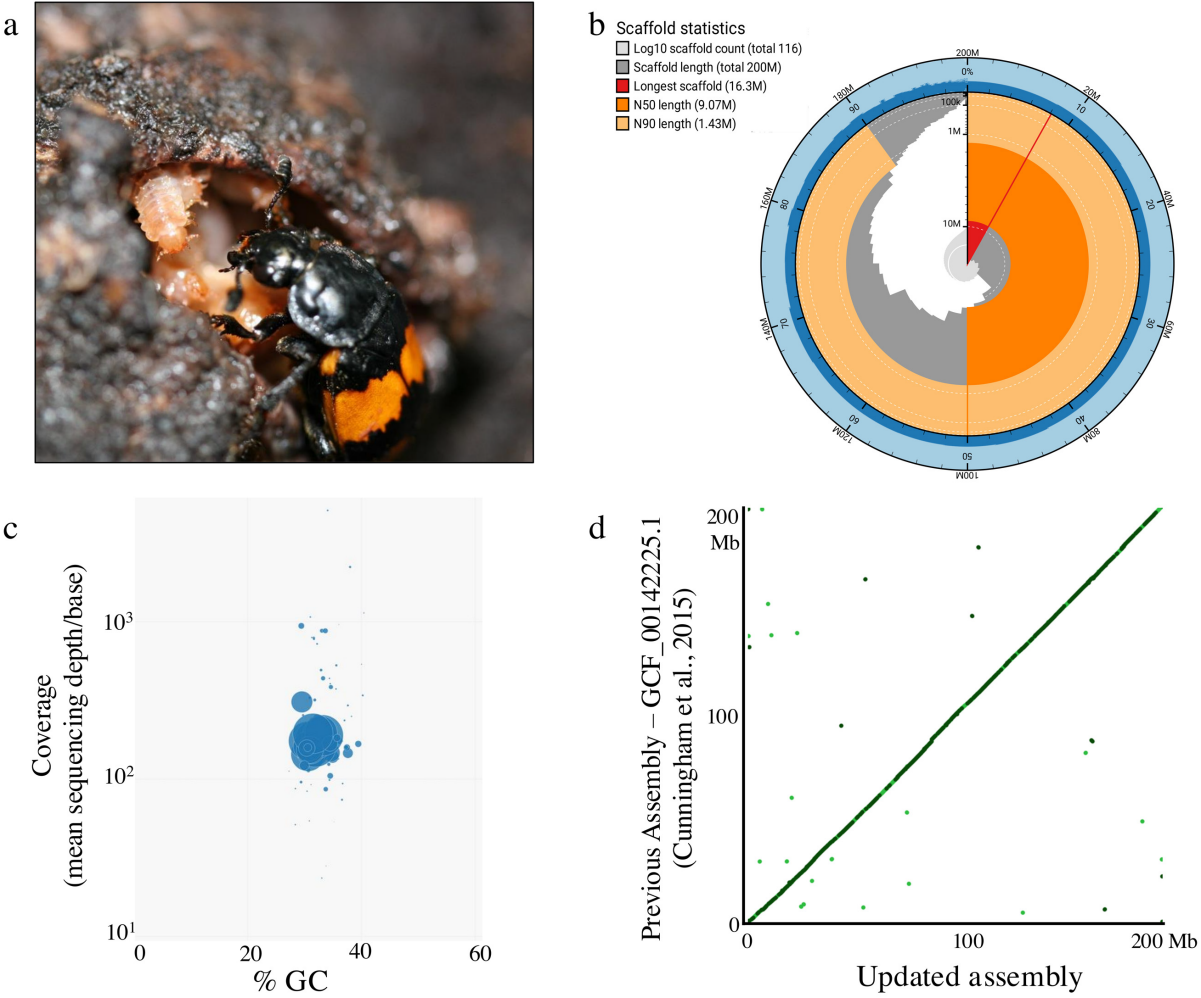
The updated genome of *Nicrophorus vespilloides* has substantially higher continuity, little contamination, and shows high sequence similarity to the previous assembly. (a) A female *N. vespilloides* pictured directly feeding a begging offspring with pre‐digested, regurgitated food. Photograph by A. J. Moore. (b) Snail plot showing the high continuity of the genome assembly, consistent GC content suggesting little contamination, and an assembly close to the predicted length of 204 Mb. Outer blue ring shows the assembly length and scaffold number, the dark blue ring shows the GC content over the assembly, the beige ring shows the N90 length and count, the orange ring shows the N50 length and count, and the gray ring shows the complete assembly in descending length of scaffolds, and the red slice shows the largest scaffold. (c) Blob plot showing the assembly scaffolds have consistent GC percentages and coverage suggesting little contamination was retained after screening. Removal of non‐Arthropoda scaffolds did not reduce BUSCO gene complement completeness. The size of the circles represents the length of the scaffold and the color represents the Order the scaffold was annotated as; blue = Arthropoda (*n* = 116). (d) Dotplot of previous assembly from Cunningham et al. (2015) mapped to the current assembly showing the high congruence of the two assemblies. Scaffolds of each genome are sorted by sequence identity with axes representing cumulative assembly length at that point. The main line is colored by the value of shared sequence identity with the darker the green representing stronger homology.

The burying beetle genus *Nicrophorus* is an ecological, evolutionary, and molecular model of complex social behavior and parental care (Eggert and Muüller 1997; Scott 1998; Cunningham 2020; Potticary et al. 2024). Beetles of this group will find small vertebrate carcasses, bury them, protect them from decomposition, and feed offspring directly through regurgitation of partially digested flesh (Figure 1a). *Nicrophorus vespilloides* is the most studied of the group, including at the molecular level. The role of neuropeptides, neurotransmitters, and immunity during the parenting of *N. vespilloides* has been examined with many genomic (Sun et al. 2020; Lewis et al. 2018; Sarkies et al. 2024), transcriptomic (Parker et al. 2015; Palmer et al. 2016; Jacobs et al. 2016; Benowitz et al. 2017; Cunningham et al. 2019), epigenetic (Cunningham et al. 2019; Lewis et al. 2020; Sarkies et al. 2024), and proteomic tools (Cunningham et al. 2017). All these studies have begun unraveling the complex transcriptional regulation of the parental care of this species. However, the next step for more refined and cell‐type specific functional genomic studies is an improved genomic resource (Pool et al. 2023; Hoedjes et al. 2024).

This resource is intended to further facilitate and improve molecular studies of this species' social behavior and parental care. This new assembly and annotation will allow *N. vespilloides* to continue to serve as a molecular model of social behavior as functional genomics techniques, such as single cell RNA‐sequencing (scRNA‐seq) or Assay of Transposon Accessible Chromatin using sequencing (ATAC‐seq) continue to improve and require ever higher quality of genomic resources. It will also provide another high‐quality genomic resource for comparative studies of beetles and insects.

## 2 Results and Discussion

### 2.1 Genome Assembly

After assembly and contamination screening, we produced an assembly of 116 scaffolds with a cumulative length of 199.97 Mb (Figure 1b,c; Table 1). This represents 97.8% of the genome's predicted size of 204 Mb based on a flow cytometry estimate (Cunningham et al. 2015). The previous assembly was 195 Mb in 4,664 scaffolds (Cunningham et al. 2015; Table 1). The new assembly increases the scaffold L/N50 to 8/9.1 Mb from 344/122 kb, while estimating the same GC content—31.8 vs. 31.9%. This represents a large improvement in the continuity of the assembly while keeping most of the previous sequence (Figure 1d). BUSCO assessed the assembly as having a near complete compliment of conserved genes (Table 1). The repeat content of the genome increased to 17.11%, compared to the previous 12.85%. The top three classifications of repeats were unclassified (9.59%), DNA elements (4.58%), and simple repeats (2.36%). The difference is likely attributable to more repetitive parts of the genome being assembled with longer reads.

**TABLE 1:** Genome assembly and annotation summary statistics for the previous and updated assemblies.

Genome			Previous assembly	Updated assembly
Scaffold number			4650	116
Contig number			8129	232
Assembly length			195	199.974 Mb
Scaffold L/N50			344/122.407 kb	8/9.069 Mb
Contig L/N50			623/67.958 kb	13/4.349 Mb
Max scaffold length			1.795 Mb	16.295 Mb
% Scaffolds > 50 kb			74.2	99.6
% GC			31.9	31.9
% Repeat			12.9	17.1
BUSCO			99.1%	99.5%
	Complete	Single Copy	97.8%	98.0%
	Complete	Duplicated	1.3%	1.5%
	Fragmented		0.5%	0.2%
	Missing		0.4%	0.3%

Annotation			Previous annotation	Updated annotation
Gene models			13,761	16,229
Protein coding			12,642	12,838
lncRNAs			983	2593
Pseudogenes			136	130
tRNAs			676	668
BUSCO			98.6%	98.7%
	Complete	Single Copy	97.4%	97.2%
	Complete	Duplicated	1.2%	1.5%
	Fragmented		0.5%	0.6%
	Missing		0.9%	0.5%

Abbreviation: BUSCO, Benchmarking Universal Single‐Copy Orthologue.

### 2.2 Genome Annotation

The previous genome annotation was 98.5% present in the new assembly (protein‐coding, tRNA's, pseudogene, lncRNA's, and miscellaneous non‐coding gene models). There were 2,973 genomic loci that did not overlap gene models from the previous annotations produced by StringTie from the mapped RNA‐seq reads. Of these, 1,871 returned BLAST hits to an insect species and were retained. Gene models that annotated to known protein‐coding (*n* = 216) or long non‐coding RNAs (*n* = 16) were retained. Gene models that did not annotate to know protein‐coding genes or long non‐coding RNAs were analyzed with CPC2 as either putative protein‐coding (*n* = 161) or putative long non‐coding RNA (*n* = 1,478) gene models. All of these novel gene models returned best BLAST hits to beetles species. This genome annotation produced a BUSCO assessment of 98.7% complete (single copy—97.2%, duplicated—1.5%), 0.6% fragmented, and 05% missing gene compliment using the most expressed transcript from every annotated locus. The finished genome annotation contains 12,838 protein‐coding and 2,593 long non‐coding RNA gene models (Table 1). All of these metrics are in line with the previous assembly and other insect genomes generally (Thomas et al. 2020), and give confidence that the new assembly and annotation are not lacking any significant biological information.

## 3 Conclusions

Understanding social behavior requires broad taxonomic sampling and resources to interrogate both the evolutionary forces acting upon it and its genetic underpinnings. Here, we presented an updated genome assembly and annotation for *Nicrophorus vespilloides* , a beetle that has elaborate parental care. The updated genome greatly improved the continuity of the assembly while retaining the near‐complete gene complement of the original assembly. This resource maintains valuable taxonomic representation for comparative studies and more refined cell‐type specific functional genomics studies moving forward.

## 4 Materials and Methods

### 4.1 Genome Assembly & Annotation

#### 4.1.1 Sequencing

We assembled 14.53 Gb of PacBio's RS II P5‐C4 CLR sequencing reads prepared at The University of Maryland's Institute for Genomic Sciences using sequencing from two SMRT cells run with a 14.4 kb and one SMRT cell run with a 15.3 kb long insert PacBio libraries (~71.2× coverage total). The first PacBio SMRT cell was also used to scaffold an Illumina assembly to produce the previous assembly (31% of the PacBio data; Cunningham et al. 2015). All data was extracted, processed, and prepared for assembly as described in Cunningham et al. (2015). This produced 7.1 Gb of error‐corrected reads using CANU (~34.8× coverage; v2.2; Koren et al. 2017).

#### 4.1.2 Assembly

Non‐default parameter values for each bioinformatic step can be found in Data S1 or mentioned here inline if we used a web‐based service. We used FLYE (v 2.9.2; Kolmogorov et al. 2019) and CANU (v 2.2; Koren et al. 2017) to generate draft assemblies. These each were scaffolded with LRScaf (v 1.1.11; Qin et al. 2019) and merged with quickmerge (v0.3; Chakraborty et al. 2016) following the program's suggested protocol. We purged haplotigs with purge_dups (v 1.2.5; Guan et al. 2020) at each step of the assembly. The assembly was then scanned for contamination with blobtools (v 1.1.1; Laetsch and Blaxter 2017) using BLAST+ (v 2.14.1; Camacho et al. 2009) against NCBI nt database (downloaded 2024‐05‐02). Scaffolds were retained if they annotated to Arthropoda. The assembly was then polished with PILON (v1.24; Walker et al. 2014) for seven rounds using the Illumina genomic reads used for the primary assembly of Cunningham et al. (2019; NCBI SRA SRX1058748) after adapter trimming using cutadapt (v4.5; Martin 2011). We also assessed each step for BUSCO completeness (v 5.5.0; Manni et al. 2021) using the Endopterygota (v.odb10) and continuity using BBMap (v 39.01; Bushnell 2014) to ensure each was producing a higher quality assembly. We used Blobtk (v0.5.3; Challis 2017) to produce the Snail plot, blobtools (v1.1.1; Laetsch and Blaxter 2017) was used to produce the Blob plot, and D‐Genies (1.5.0; Cabanettes and Klopp 2018; non‐default parameter: sorted, hide noise selected, 67% value of filter small matches) was used to produce the dotplot of the previous and current assembly.

#### 4.1.3 Repeats

We used RepeatModeler (v2.0.4; Flynn et al. 2020) to identify and classify the repetitive elements of the assembled genome. RepeatMasker (v4.1.4; Smit, Hubley, and Green 2013–2015) was then used to mask the genome and produce repetitive element content estimates.

#### 4.1.4 Annotation

We used Liftoff (v 1.6.3; Shumate and Salzberg 2021) to map the current NCBI RefSeq Annotation (Accession *Nicrophorus vespilloides* Annotation Release 100) of the *N. vespilloides* genome (NCBI Accession GCF_001412225.1) to the new assembly. In addition, we produced new gene annotations from RNA‐seq data. We used FLASH (v 2.2.0; Magoc and Salzberg 2011), HISAT2 (v 2.2.1; Kim et al. 2019), and StringTie (v 2.2.1; Pertea et al. 2015) to assemble new gene models using RNA‐seq data after quality control with cutadapt from Parker et al. (2015), Palmer et al. (2016), Jacobs et al. (2016), Benowitz et al. (2017), and Cunningham et al. (2019). The gene models generated were compared to the annotation that was mapped from the previous annotation. Any genomic loci that were identified that were not overlapping with any gene model from the previous annotation that recovered BLAST hits from NCBI's nt database (−evalue 1*e*‐30) that annotated to an insect species was retained. If the gene model was annotated to a known protein‐coding or long non‐coding RNA, it was annotated as such. The remaining gene models were analyzed with CPC2's webserver using default settings (vBeta; Kang et al. 2017). We assessed the completeness of the final annotation using BUSCO with the Endopterygota (v.odb10) dataset using the highest expressed isoform from each locus measured by TPM.

## Author Contributions

**Christopher B. Cunningham:** conceptualization (lead), data curation (lead), formal analysis (lead), investigation (lead), methodology (lead), project administration (lead), visualization (lead), writing – original draft (lead), writing – review and editing (lead). **Kyle M. Benowitz:** conceptualization (supporting), formal analysis (supporting), investigation (supporting), methodology (supporting), visualization (supporting), writing – review and editing (equal). **Allen J. Moore:** conceptualization (equal), funding acquisition (equal), methodology (equal), supervision (equal), writing – review and editing (equal).

## Conflicts of Interest

The authors declare no conflicts of interest.

## Supporting information


Data S1.

## Data Availability

The raw and processed data that support the findings of this study are openly available in under NCBI BioProject PRJNA1123269. This genomic resource is also available at the USDA's National Agricultural Library i5k Workspace.
